# Downstream Signaling of Inflammasome Pathway Affects Patients’ Outcome in the Context of Distinct Molecular Breast Cancer Subtypes

**DOI:** 10.3390/ph15060651

**Published:** 2022-05-24

**Authors:** Concetta Saponaro, Annarita Fanizzi, Margherita Sonnessa, Paolo Mondelli, Daniele Vergara, Donato Loisi, Raffaella Massafra, Agnese Latorre, Francesco A. Zito, Laura Schirosi

**Affiliations:** 1 Pathology Department, IRCCS Istituto Tumori “Giovanni Paolo II”, 70124 Bari, Italy; c.saponaro@oncologico.bari.it (C.S.); m.sonnessa@oncologico.bari.it (M.S.); p.mondelli@oncologico.bari.it (P.M.); d.loisi@oncologico.bari.it (D.L.); a.zito@oncologico.bari.it (F.A.Z.); 2 Department of Health Physics, IRCCS Istituto Tumori “Giovanni Paolo II”, 70124 Bari, Italy; a.fanizzi@oncologico.bari.it (A.F.); r.massafra@oncologico.bari.it (R.M.); 3 Department of Biological and Environmental Sciences and Technologies (DiSTeBA), University of Salento, 73100 Lecce, Italy; daniele.vergara@unisalento.it; 4 Medical Oncology Unit, IRCCS Istituto Tumori “Giovanni Paolo II”, 70124 Bari, Italy; a.latorre@oncologico.bari.it

**Keywords:** inflammasome NLRP3, PYCARD, CyclinD1, MYC, *CCND1*, *MYC*, Breast Cancer

## Abstract

Inflammasomes are protein complexes involved in the regulation of different biological conditions. Over the past few years, the role of NLRP3 in different tumor types has gained interest. In breast cancer (BC), NLRP3 has been associated with multiple processes including epithelia mesenchymal transition, invasion and metastization. Little is known about molecular modifications of NLRP3 up-regulation. In this study, in a cohort of BCs, the expression levels of NLRP3 and PYCARD were analyzed in combination with CyclinD1 and *MYC* ones and their gene alterations. We described a correlation between the NLRP3/PYCARD axis and CyclinD1 (*p* < 0.0001). NLRP3, PYCARD and CyclinD1’s positive expression was observed in estrogen receptor (ER) and progesterone receptor (PgR) positive cases (*p* < 0.0001). Furthermore, a reduction of NLRP3 and PYCARD expression has been observed in triple negative breast cancers (TNBCs) with respect to the Luminal phenotypes (*p* = 0.017 and *p* = 0.0015, respectively). The association NLRP3+/*CCND1+* or PYCARD+/*CCND1+* was related to more aggressive clinicopathological characteristics and a worse clinical outcome, both for progression free survival (PFS) and overall survival (OS) with respect to NLRP3+/*CCND1−* or PYCARD+/*CCND1−* patients, both in the whole cohort and also in the subset of Luminal tumors. In conclusion, our study shows that the NLRP3 inflammasome complex is down-regulated in TNBC compared to the Luminal subgroup. Moreover, the expression levels of NLRP3 and PYCARD together with the alterations of *CCND1* results in Luminal subtype BC’ss poor prognosis.

## 1. Introduction

Breast cancer (BC) is the most common type of cancer in women, and metastatic cancers are the most critical forms of related death [1]. In 2020, there were 54,976 new diagnoses of BC in Italy. This represents 30% of all female diagnosed cancers [2]. Different biological processes contribute to invasive phenotypes of BC, and between them a pivotal role is played by inflammation, which increases the production of growth factors, free radicals, prostaglandins and the pro-inflammatory cytokines [interleukin (IL)-1β and IL-18] [3]. In this scenario, the main actors are inflammasome complexes. Inflammasomes are cytosolic protein complexes that are assembled in response to microbial infection and cellular damage. The inflammasome platform classically comprises a sensor of a NOD-like receptor protein (NLRP), the adaptor molecule apoptosis-associated speck-like protein, which is characterized by two domains: Pyrin and CARD (ASC) and a procaspase domain. ASC is also known as PYCARD. Homotypic CARD-CARD or PYD-PYD connections are needed for inflammasome assembly and interaction with caspases [4]. After inflammasome activation, a series of proteolytic cleavages trigger the formation of mature and biologically active inflammatory interleukins (such as IL-1β and IL-18) [5]. NLRP3 inflammasome complex is the most intensively investigated one, given its possible contribution in several human diseases, including immune, inflammatory and cancer diseases [6,7,8,9,10,11]. During the last years, its role in BC has been fully debated and, above all, its involvement in the epithelia mesenchymal transition (EMT), in the promotion of migration, invasion and metastization of BC cells. Wang Y. and colleagues reported an increase of metastatic conditions through the up-regulation of the NLRP3-IL-1β axis [12]. The down regulation of NLRP3 inflammasome by Raloxifene, a selective estrogen receptor (ER) modulator, was also reported in relation to the inhibition of BC growth, demonstrating its involvement during cancerogenesis and its functional connection with hormone status [13]. Our previous study demonstrated the overexpression of NLRP3 in the tumor compartment compared to the non-tumor area in primary invasive BCs and its impact on disease free survival (DFS). In fact, high NLRP3 expression was associated with a worst DFS in this BC cohort [14]. Inflammasome crosses different pathways involved in cancer progression, including the Wnt/β-catenin/CyclinD1 signaling. β-catenin was reported to participate in several inflammatory diseases [15,16,17,18,19] and its role in cancerogenesis is well known. Different studies demonstrated that Wnt/β-catenin/CyclinD1 signaling pathway is crucial for metastasis regulation, cell proliferation and EMT in BC [20,21,22,23].

In a non-small cell lung cancer model, the use of eat-clearing and detoxifying herbs reduced NLRP3 and CyclinD1 expression, causing cellular proliferation and a decrease in tumor growth [24]. Furthermore, in a gastric cancer (GC) model, NLRP3 promoted CyclinD1 gene (*CCND1*) transcription, enhancing GC proliferation and tumorigenesis [25]. 

NLRP3 was also associated with the increase of migration and invasion of colorectal cancer cells in an inflammatory microenvironment and with lymphoma cells proliferation and apoptosis inhibition, through the up-regulation of *MYC* and Bcl2 [26,27]. The inflammatory cytokine IL-1β may have a role in the promotion of *CCND1* and *MYC* expression through the stabilization of β-catenin and its nuclear translocation [28].

The possible involvement of *CCND1* and *MYC* as inflammasome downstream targets in BC is unknown, so the aim of our study was to elucidate the downstream signaling pathway triggered by the NLPR3 inflammasome activation in different BC molecular subtypes in order to discover novel specific potential prognostic biomarkers and identify specific subgroups of patients who could be directed to novel and more specific target therapies.

## 2. Results

### 2.1. Protein and Gene Status Profiling

**Figure 1:**
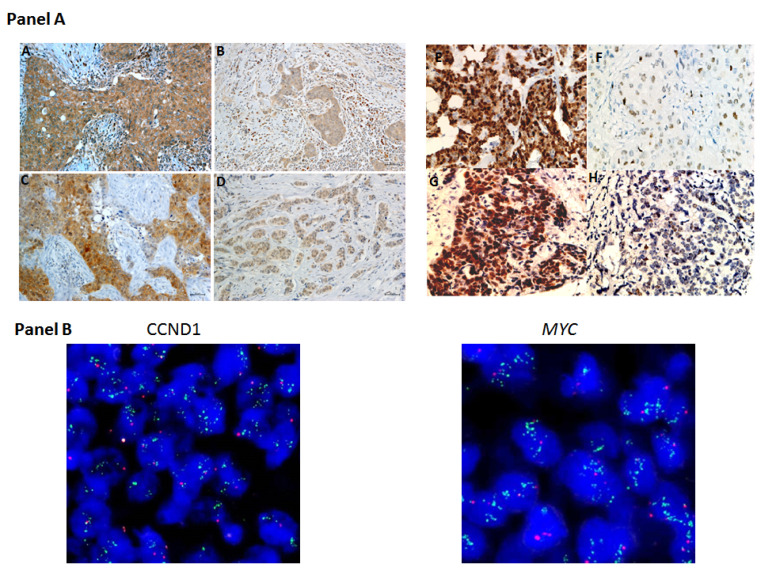
Representative images of immunohistochemical staining in BC tissues. **Panel A** displays representative images of biomarker expression. On the left: (**A**) Positive NOD-like receptor protein 3 (NLRP3) expression, (**B**) negative NLRP3 expression, (**C**) Positive Apoptosis-Associated Speck-Like Protein Containing a Pyrin and CARD domain (PYCARD) expression, (**D**) Negative PYCARD expression; On the right: (**E**) Positive CyclinD1 expression, (**F**) negative CyclinD1 expression, (**G**) Positive MYC expression, (**H**) Negative MYC expression. **Panel B** displays representative images of FISH experiments: (**on the left**) *CCND1* gene amplification; (**on the right**) *MYC* gene amplification. For immunohistochemistry: original magnification, ×400. Scale bar = 20 μm.

High NLRP3 and PYCARD expression was found in 50% (113/225) and 31% (68/222) of analyzed BCs. High CyclinD1 and MYC protein expression was found in 48% (109/229) and 32% (74/232), of tested samples, respectively. Furthermore, the fluorescence in situ hybridization (FISH) analysis showed *CCND1* and *MYC* gene alterations in the 30% (46/152) and 40% (59/148) of cases, respectively (Table 1). Figure 1 panel A shows examples of the staining pattern of the proteins analyzed by immunohistochemistry (IHC), and Figure 1 panel B shows examples of *CCND1* and *MYC* gene amplification.

### 2.2. Relationship between Tumor Marker Alterations and Clinicopathological Characteristics

Table 2 shows the relationship between protein expression (NLRP3, PYCARD, CyclinD1 and MYC) and *CCND1* and *MYC* gene alterations and the clinicopathological characteristics. NLRP3 over-expression was observed in invasive ductal carcinoma (IDC; *p* = 0.012) and in ER and progesteron receptor (PgR) positive cases (*p* < 0.0001 for both). PYCARD high expression was also related to ER and PgR positive status (*p* < 0.0001 and *p* = 0.003, respectively). CyclinD1 positive expression was observed in older women (*p* = 0.039) and it was also associated with ER and PgR positive status (*p* < 0.0001 for both). MYC positive expression was observed in younger women (*p* = 0.039), in IDCs (*p* = 0.035) and in the cases with a high proliferative activity (Ki67) (*p* = 0.0047). MYC was also inversely related to ER status (*p* = 0.033). *CCND1* gene alterations were associated with high Ki67 (*p* = 0.064). *MYC* gene alterations confirmed the positive relationship with high Ki67 (*p* = 0.0004) and the negative association with ER (*p* = 0.0002). Furthermore, it was also inversely related to PgR (*p* = 0.0027) and its alterations were associated with G3 cancers (*p* < 0.0001).

### 2.3. Biomarker Assessment in Relation to Molecular Phenotype

**Figure 2:**
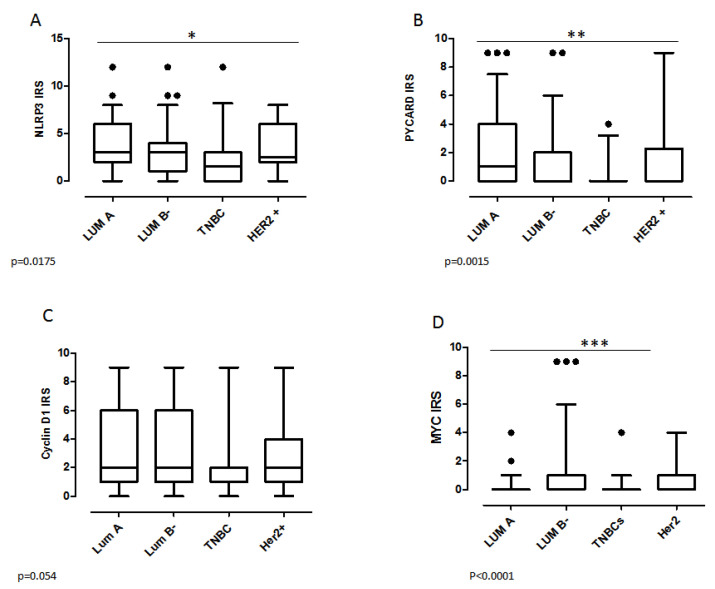
Protein expression level in the different molecular BC subgroups. (**A**) NLRP3 expression. (**B**) PYCARD expression. (**C**) CyclinD1 expression and (**D**) MYC expression by Anova test. IRS: Immuno Reactive Score. The IRS was calculated by the product of the proportional score (PS) obtained from the percentage of positive cells and the intensity of the staining (see materials and methods section). Values are expressed as a median (horizontal bold line in each box), dot indicates outliers. * *p* < 0.05; ** *p* < 0.01; *** *p* < 0.001.

We also investigated the protein expressions and gene alterations in the different molecular BC subgroups. In our cohort, 32% (75/237) of cases were Luminal A, 38% (89/237) were Luminal B−, 13% (32/237) were human epidermal growth factor receptor 2 positive (HER2+) and 17% (41/237) were triple negative breast cancers (TNBCs); for three cases it wasn’t possible to attribute the molecular subtype. In the TNBC subgroup, the χ2 analysis revealed a clear association with the negative NLRP3, PYCARD, CyclinD1 and MYC status (*p* = 0.01, *p* = 0.052, *p* = 0.003 and *p* = 0.01, respectively) (Table 2). On the contrary, a positive expression of PYCARD was observed in the Luminal A subtype (*p* = 0.009), while an inverse relation was found between MYC expression and Luminal A (*p* = 0.01); furthermore, a positive MYC expression was associated with the Luminal B− phenotype (*p* < 0.0001). The great majority of samples in the Luminal A subgroup also had a normal status of *CCND1* and *MYC* (*p* = 0.02 and *p* = 0.001, respectively). These data were confirmed by Anova test. NLRP3 and PYCARD expressions were lower in TNBCs with respect to Luminal phenotype (*p* = 0.017 and *p* = 0.0015 respectively) (Figure 2A,B). MYC expression was also reduced in Luminal A and TNBCs with respect to Luminal B (Figure 2D), while no statistically significant variation was found for CyclinD1 expression (Figure 2C).

### 2.4. Correlation between Analyzed Protein Expressions

**Figure 3:**
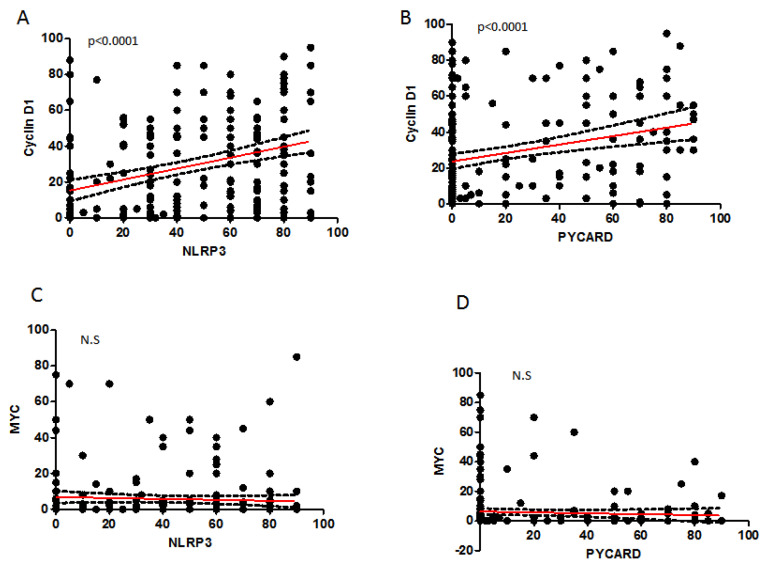
Spearman correlation (SC) between proteins. (**A**) SC between NLRP3 and CyclinD1. (**B**) SC between PYCARD and CyclinD1. (**C**) SC between NLRP3 and MYC. (**D**) SC between PYCARD and MYC. NS: not significant.

The Spearman correlation test on continuous variables, showed a statistically significant direct relation of CyclinD1 with NLRP3 (r: 0.366; *p* < 0.0001), and with PYCARD expression (r: 0.285; *p* < 0.0001) (Table 3 and Figure 3). In the other cases, we did not observe any significant correlation. 

### 2.5. Relationship between Coupled Biomarkers and Clinicopathological Characteristics

**Figure 4:**
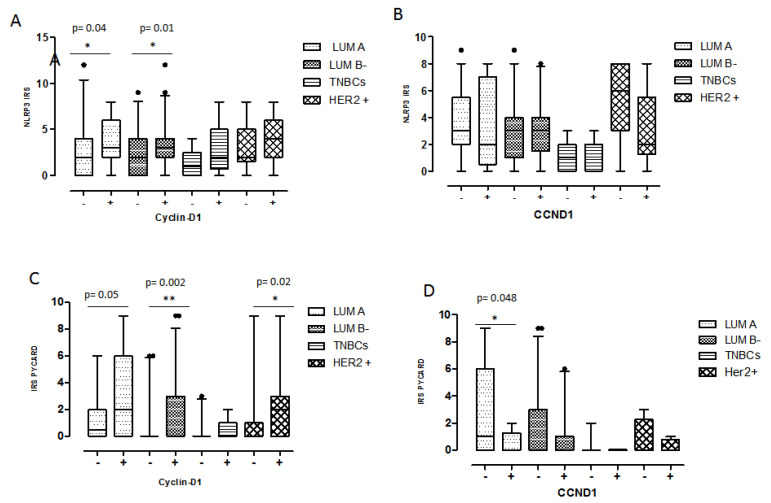
The distribution analysis of CyclinD1 and *CCND1* negative and positive cases with respect to the inflammasome proteins in the different molecular groups. (**A**) NLRP3 expression in CyclinD1 positive and negative cases. (**B**) NLRP3 expression in *CCND1* positive and negative cases. (**C**) PYCARD expression in CyclinD1 positive and negative cases. (**D**) NLRP3 expression in *CCND1* positive and negative cases. IRS: Immuno Reactive Score. The IRS was calculated by the product of the Proportional Score (PS) obtained from the percentage of positive cells and the intensity of the staining (see the materials and methods section). Values are expressed as a median (horizontal bold line in each box), dot indicates outliers. * *p* < 0.05; ** *p* < 0.01.

To better investigate a possible interaction of CyclinD1 and the inflammasome pathway, we studied the association of NLRP3-/CyclinD1- and NLRP3+/CyclinD1+ phenotypes versus the clinicopathological features. A higher expression of NLRP3 and CyclinD1 (NLRP3+/CyclinD1+) was associated with older patients (*p* = 0.0289), with positive ER and PgR expression (*p* = 0.0016 and *p* = 0.026). Taking into account, instead, the interaction of CyclinD1 and PYCARD, a higher expression of them (PYCARD+/CyclinD1+) was associated with positive ER expression as well (*p* = 0.0017), and to higher Ki67 expression (*p* = 0.032) (Table 4A). The analysis was also performed considering both the inflammasome proteins in combination with *CCND1* gene alterations with respect to the clinicopathological features. In detail, the only significant result was the statistical association between PYCARD+/*CCND1+* cases and the positive node status (*p* = 0.033) (Table 4A). The distribution analysis of CyclinD1 and *CCND1* negative and positive cases with respect to the inflammasome proteins in the different molecular groups revealed a higher NLRP3 expression in CyclinD1 positive cases in the Luminal phenotype (Luminal A *p* = 0.04 and Luminal B- *p* = 0.01) (Figure 4A). No statistically significant differences were observed with respect to *CCND1* (Figure 4B). PYCARD was more expressed in CyclinD1 positive cases in the Luminal B- (*p* = 0.002) and HER2+ subgroups (*p* = 0.02) (Figure 4C). Regarding PYCARD and *CCND1* gene alteration, we observed a slightly higher PYCARD positivity in *CCND1* negative Luminal A cases (*p* = 0.048) (Figure 4D).

**Figure 5:**
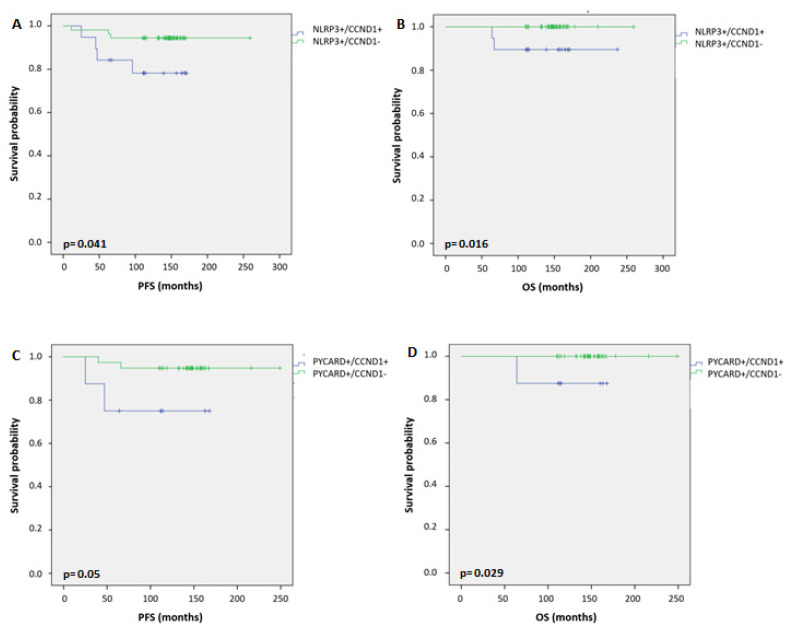
Kaplan–Maier curve analysis. (**A**) Kaplan–Maier curve for progression-free survival (PFS) according to NLRP3+/*CCND1+* versus NLRP3+/*CCND1−* patients (*p* = 0.041). (**B**) Kaplan–Maier curve for overall survival (OS) according to NLRP3+/*CCND1+* versus NLRP3+/*CCND1−* patients (*p* = 0.016). (**C**) Kaplan–Maier curve for PFS according to PYCARD+/*CCND1+* versus PYCARD+/*CCND1−* patients (*p* = 0.05). (**D**) Kaplan–Maier curve for OS according to PYCARD+/*CCND1+* versus PYCARD+/*CCND1−* patients (*p* = 0.029).

Univariate analysis (Table 5) demonstrated that PYCARD overexpression was associated with a long PFS (*p* = 0.017), while with respect to OS we observed only a trend (*p* = 0.059). *MYC* gene alteration was a significant risk factor for unfavorable PFS (*p* = 0.017). MYC protein expression and *CCND1* gene alteration were both significant risk factors for a worse OS (*p* = 0.041 and *p* = 0.013, respectively). Kaplan-Meier curves confirmed what was found in the univariate analysis (Figure S1, Supplementary Materials). In order to evaluate the impact of the association between *CCND1* gene alteration and the protein inflammasome proteins on the prognosis, we performed Kaplan-Meier curves which revealed that BC patients with positive NLRP3 expression and contextually *CCND1* alteration (NLRP3+/*CCND1+*) had a worse PFS (*p* = 0.041, Figure 5A) and OS (*p* = 0.016, Figure 5B) compared to patients with positive NLRP3 expression but without *CCND1* gene alteration (NLRP3+/*CCND1−*. Similar results were observed considering the interaction of PYCARD with *CCND1*. Patients with PYCARD+/*CCND1+* showed a worse PFS (*p* = 0.052, Figure 5C) and OS (*p* = 0.029, Figure 5D) compared to patients with PYCARD+/*CCND1−*. Taking into account the subgroup of Luminal tumors (Luminal A and B), we found the same results only for patients with NLRP3+/*CCND1+* phenotype with respect to those with NLRP3+/*CCND1−*: they showed a worse PFS and OS (*p* = 0.011 and *p* = 0.015, respectively; Figure S1). The patients with PYCARD+/*CCND1+* versus PYCARD+/*CCND1−* phenotype showed a worse PFS, even though it was not statistically significant (*p* = 0.062), and a shorter OS (*p*= 0.034) (Figure S2, Supplementary Materials). A multivariate analysis (Table 6) showed that *MYC* gene amplification was an independent risk factor for PFS (*p* = 0.036). Additionally, no factors turned a significant difference.

## 3. Discussion

The NLRP3 inflammasome complex plays a key role during the development of various human malignancies, including different cancer types [29,30,31]. For this reason, NLRP3 could be a good candidate as a prognostic and therapeutic biomarker. The NLRP3 platform is an intricate network that is able to trigger different downstream signaling targets [32]. In this study we show a strong correlation between NLRP3 and CyclinD1 and their synergic action is able to affect patients’ clinical outcome. 

Our recent data demonstrated a higher NLRP3 and PYCARD expression in the tumor compartment with respect to the non-tumor areas in a BC cohort [14]. Here, we confirm the high NLRP3 and PYCARD expression in tumor samples (50% and 31%, respectively) as well as an overexpression of CyclinD1 and MYC, (48% and 32%, respectively). Furthermore, in our cohort we also observed 30% of cases and 40% of cases with *CCND1* and *MYC* gene alterations, respectively. Anomalous NLRP3 expression was reported in different tumors [8,9,33,34], and it’s up-regulation in breast cancer-associated fibroblasts (CAFs) is related to cancer progression and metastasis [35]. A strong correlation of NLRP3, PYCARD and CyclinD1 with hormone status was observed in our samples, suggesting a possible association with the Luminal phenotype. These data are also supported by the overexpression of NLRP3 and PYCARD in CyclinD1 positive cases in the Luminal subgroups. The increased expression of NLRP3 actives IL-1β, which, through its Toll/interleukin-1 receptor (TIR) domains, induces β-catenin nuclear translocation and *MYC* and *CCDN1* activation [28]. Different studies have reported the regulation of NLRP3 by the ER trigging pathway involved in cancer progression as the Wnt/β-catenin/CyclinD1 signaling pathway [36,37,38,39]. ER is also involved in endometrial cancer cell proliferation through the up-regulation of NLRP3 expression [36]. In order to clarify this point, we examined the NLRP3 expression in relation to different BC molecular phenotypes. Both NLRP3 and PYCARD showed a significant reduction of expression in the TNBC subgroup, underlining the key role of the inflammasome platform in the Luminal phenotype. To the best of our knowledge, this is the first study that demonstrated a down-regulation of the NLRP3 inflammasome in human TNBC samples, indirectly confirming the involvement of hormonal receptors for inflammasome activation, as previously reported by different authors [36,37,38].

A Spearman correlation showed a strong association between CyclinD1 and NLRP3 and PYCARD, giving important information about a possible downstream mechanism of activation. A recent study reports that the Angiopoietin-like 2 knockdown inhibited the activation of the NLRP3 inflammasome and it down-regulated CiclinD1 protein expression [40]. In a pancreatic cancer model, PYCARD silencing inhibited the cell growth, reducing CyclinD1 levels as well [41]. 

Our results fit in this wake, linking the activation of the inflammasome platform to the specific CyclinD1 downstream signaling. Furthermore, the association of NLRP3+/CyclinD1+ or with *CCND1+* and PYCARD+/CyclinD1+ or with *CCND1+* was related to more aggressive clinicopathological characteristics. These data were confirmed by the survival analysis, where Kaplan-Meier curves showed a worse clinical outcome, both for PFS and OS, in the subgroup of patients with overexpression of NLRP3 or PYCARD and *CCND1* alterations, both in the whole cohort and also in the subgroup of Luminal tumors. These results underline the finding that when this inflammasome complex abnormally triggers the activation of the *CCND1* pathway, it affects the survival of patients with a Luminal phenotype. The ability of NLRP3 to bind to the *CCND1* promoter and to support its transcription was already described in gastric cancer [25]. For the first time, these data demonstrate that in some BC patients the downstream signaling of inflammasome proteins involves *CCND1* activation. Analyzing the single Kaplan-Meier curve, PYCARD was more expressed in the subgroup of patients with a better PFS and OS. Differently, the subgroup with *MYC* and *CCND1* gene alterations and with high MYC expression showed a worse PFS and OS. PYCARD is an important adaptor molecule that is necessary for inflammasome platform activation and a pro-apoptotic molecule trigger. It’s down-regulation, by aberrant methylation, was often associated with different tumor types. Its silencing is an epigenetic alteration that can affect its suppressor activity by apoptosis inhibition [42,43,44,45]. In pancreatic ductal adenocarcinoma (PDAC) cells, PYCARD silencing affects CyclinD1, blocking the cell cycle at the G1 phase. These data suggest an inhibitory role of PYCARD during cancer progression, thus supporting its potential therapeutic role in cancer [40]. In our cohort, high PYCARD expression, linked to a better patient outcome, could be the sum of a high presence of the Luminal subtype with a better outcome and a lesser presence of aberrant methylation which could prevent apoptotic events. These contradictory data suggest that the role of PYCARD in BC could depend on the pathological tumor type, stage, molecular profile and tumor microenvironment. *CCND1* and *MYC* gene alterations as well as MYC overexpression were associated with a worse outcome. Their close relationship with cancer progression has been confirmed by different authors [46,47,48,49,50]. 

To further support the oncogenic role of *MYC*, a multivariate analysis demonstrated that *MYC* gene alterations were an independent prognostic factor associated with a shorter PFS. 

## 4. Materials and Methods

### 4.1. Patients and Clinicopathological Characteristics

A retrospective, non-consecutive series of 240 patients with confirmed primary invasive BC from the Istituto Tumori “Giovanni Paolo II” of Bari, Italy, were included in this study. The patients were selected on the availability of biological material and their clinical follow-up. Patients were eligible if they had a histological diagnosis of invasive BC of any size and no evidence of metastatic disease at diagnosis. The study was approved by the Ethics Committee of the Istituto Tumori “Giovanni Paolo II” with document no. 234/CE of 13 November 2017. Table 1 summarizes the clinicopathological characteristics of the entire cohort. The median age was 53 years. The tumor, node, metastasis (TNM) classification, tumor size, histological grade, ER status, PgR status, proliferative activity (Ki67) expression and HER2 status were provided by the Pathology Department of our Institute. The immunohistochemical assessment of ER status, PgR status and Ki67 expression were previously reported [51]. Cases scoring 0 and 1+ were classified as negative. HER2 was considered to be positive if immunostaining was 3+ or if a score of 2+ showed gene amplification by FISH, according to the 2018 ASCO/CAP guidelines for BC [52]. The molecular subtype was assigned according to the WHO 2019 guidelines for BC. Specifically, the cases were considered Luminal A with ER ≥ 1%, PgR ≥ 1% and Ki67 < 14%; Luminal B- with ER ≥ 1%, PgR ≥ 1%, Ki67 ≥ 14% and HER2 negative; TNBCs with ER < 1%, PR < 1% and HER2 negative. Our HER2 group consisted of all HER2 positive cases.

### 4.2. Tissue Microarrays and Immunohistochemistry

Tissue microarrays (TMAs) were prepared and IHC was performed as previously reported [14]. Consecutive sections of 4-µm thickness were cut from formalin-fixed and paraffin-embedded histological material and stained with an indirect immunoperoxidase method using the BenchMark XT automated staining instrument (Ventana Medical Systems, Tucson, AZ, USA) for NLRP3 and PYCARD, [14]. Antigen retrieval was made with Cell Conditioning solution 1 at 95° for NLRP3 (32 min), and Cell Conditioning solution 2 at 95 °C for PYCARD (32 min). The slides were then incubated at 37° for 1 h with the specific primary antibodies as reported in Table S1, Supplementary Materials. The OptiView DAB IHC Detection Kit and OptiView Amplification Kit (Ventana Medical Systems, Tucson, AZ, USA) were used to detect NLRP3 and PYCARD protein expression. Finally, tissues were counterstained with hematoxylin and a bluing reagent for 8 min and 4 min, respectively, and were then dehydrated and mounted.

CyclinD1 and MYC staining were performed using the OMNIS DAKO platform (Dako, CA, USA) with the specific primary antibodies as reported in Table S1, according to the manufacturer’s instructions. Positive and negative controls were included in each staining run as indicated in the datasheet of each antibody. All of the antibodies used in this study were validated in the pre-analytic phase to guarantee a satisfactory level of reproducibility and accuracy. All of the solutions were from Ventana Medical Systems unless otherwise specified.

### 4.3. Immunohistochemical Assessment

The cytoplasmic expression of NLRP3, PYCARD and the nuclear expression for CyclinD1 and MYC were considered. Sections were examined under light microscope (Zeiss, Oberkochen, Germany) and assessed by an immunoreactive score (IRS). The IRS was calculated by the product of the percentage of positive cells and the intensity of the staining. For NLRP3 the percentage of positive cells (4, ≥76%; 3, 51–75%; 2, 26–50%; 1, 1–25%; 0, 0%) and the intensity of the staining (3, strong; 2, moderate; 1, mild; and 0, no staining) resulted in IRS scores between 0 (no staining) and 12 (maximum staining). For PYCARD the percentage of positive cells (3, 67–100%; 2, 34–66%; 1, ≤33%; 0, 0%) and the intensity of the staining (3, strong; 2, moderate; 1, mild; and 0, no staining) resulted in IRS scores between 0 (no staining) and 9 (maximum staining). For CyclinD1 and MYC the percentage of positive cells (3, 51–100%; 2, 26–50%; 1, ≤25%; 0, 0%) and the intensity of the staining (3, strong; 2, moderate; 1, mild; and 0, no staining) resulted in IRS scores between 0 (no staining) and 9 (maximum staining).

All stained specimens were assessed independently by two observers who were blinded to the clinicopathological data. Three distinct visual fields were selected to evaluate the slides using x400 magnification. Discordant scores were reviewed and resolved by discussion.

### 4.4. Detection of CCND1 and MYC Gene Alterations by FISH

The 3-µm thick TMA sections were tested by FISH for the detection of *CCND1* and *MYC* gene alterations as amplification, using *ZytoLight SPEC CCND1/CEN11* and *ZytoLight SPEC MYC/CEN11* probes (ZytoVision), respectively. The sections were processed using the FISH-Tissue Implementation kit (ZytoVision) according to the manufacturer’s instructions. The analysis of samples was made using a Leica DM5500B fluorescence microscope (Leica) with a 60X objective and CytoVision v.7.4 (Leica) image analysis software. Each spot was scored, counting at least 20 tumor cells and then a sum was made of the cells counted for each case. The median signal number for the gene and centromere copies was recorded in order to calculate the ratio. A case was considered as “amplified” if the ratio was ≥2 and the gene copy number ≥4, “polisomic” if the ratio was <2 with a gene copy number in the range 2.5–6 and a centromere copy number ≥2.5, and “amplified and polisomic” if the gene copy number was ≥6 and the centromere copy number was ≥2.5. To perform the statistical analysis, positive cases were those with any alteration (amplified, polisomic or both) while the others were considered as negative. FISH evaluation was performed by two biologists, independently. Discordant cases were reviewed and resolved by discussion.

### 4.5. Follow-Up and Statistical Analysis

Progression-Free Survival (PFS) was defined as the time from the date of surgery to the date of first relapse or progression of disease or to the date of a second invasive BC/secondary primary cancer and/or death without evidence of BC or to the date of the last follow-up. Overall Survival (OS) was defined as the time between the date of surgery and the date of death from any cause or the date of the last follow-up.

Time-to-event variables were estimated using the Kaplan-Meier method and comparisons between curves were done using the Log-rank test.

In order to identify the prognostic factors for DFS and OS, univariable and multivariable Cox regression models were used to estimate hazard ratios (HR) and their 95% confidence intervals (95% CI).

The association of baseline factors and protein expressions was evaluated with a Chi-square test, while the correlation between continuous variables was evaluated with a Spearman correlation test. The biomarkers expression in the different molecular subgroups was evaluated with a Kruskal-Wallis test.

Statistical analyses were performed using the Prism version 5.00 software package (Graph-Pad Software, San Diego, CA, USA) and SPSS Statistics version 22.0 (IBM). *p* < 0.05 was considered to be statistically significant.

## 5. Conclusions

In conclusion, our study reveals that the NLRP3 inflammasome complex is down-regulated in TNBCs compared to the Luminal BC phenotype; *MYC* gene alterations are an independent prognostic factor for PFS and we highlight, for the first time, that the combination axis of NLRP3+/*CCND1+* and PYCARD+/*CCND1+* is associated with a poor-clinical outcome of BC patients in the Luminal subtype also.

In summary, our study provides novel insights into the NLRP3 inflammasome downstream signaling in combination with *CCD1* activation and alterations in BC patients and in particular in the Luminal tumor phenotype. These findings have important implications for the identification of novel clinical prognostic biomarkers and novel possible therapeutic targets.

## Figures and Tables

**Table 1:** Tumor characteristics of 240 invasive breast cancer patients.

	N. (%)
**Age** (**years**): median value (range 29–80)	53
≤53	121 (50)
>53	119 (50)
**Histotype**	
IDC	205 (86.0)
ILC	17 (7.0)
Other	17 (7.0)
unknown	1
**Tumor size** (**cm**)	
≤2.0	137 (58)
>2.0	99 (42)
Unknown	4
**Node**	
Negative	149 (63)
Positive	86 (37)
unknown	5
**Grade**	
1	18 (8)
2	107 (45)
3	113 (47)
unknown	2
**ER** (**%**)	
<1	85 (36)
≥1	152 (64)
unknown	3
**PgR** (**%**)	
<1	100 (42)
≥1	137 (58)
unknown	3
**Ki67** (**%**)	
<14	83 (35)
≥14	154 (65)
unknown	3
**HER2**	
Negative	204 (86)
Positive	33 (14)
unknown	3
**Molecular Subtype**	
Luminal A	75 (32)
Luminal B−	89 (38)
Her2+	32 (13)
TNBC	41 (17)
unknown	3
**NLRP3**	
Negative	112 (50)
Positive	113 (50)
unknown	15
**PYCARD**	
Negative	154 (69)
Positive	68 (31)
unknown	18
**CyclinD1**	
Negative	120 (52)
Positive	109 (48)
unknown	11
**MYC**	
Negative	158 (68)
Positive	74 (32)
unknown	8
***CCND1***	
Negative	106 (70)
Positive	46 (30)
unknown	88
***MYC***	
Negative	89 (60)
Positive	59 (40)
unknown	92

IDC: Invasive ductal carcinoma; ILC: Invasive lobular carcinoma; ER: Estrogen receptor; PgR: Progesterone receptor; HER2: Human epidermal growth factor receptor 2; NLRP3: NOD-like receptor protein 3; PYCARD: Apoptosis-Associated Speck-Like Protein Containing a Pyrin and CARD domain; *CCND1*: Cyclin D1 gene; MYC protein; *MYC* gene.

**Table 2:** Relationship between tumor markers and clinicopathological features.

	Protein Expression												*Gene Alteration*					
	NLRP3			PYCARD			Cyclin D1			MYC			*CCND1*			*Myc*		
	Negative	Positive		Negative	Positive		Negative	Positive		Negative	Positive		Negative	Positive		Negative	Positive	
	N (%)	N (%)	*p*	N (%)	N (%)	*p*	N (%)	N (%)	*p*	N (%)	N (%)	*p*	N (%)	N (%)	*p*	N (%)	N (%)	*p*
**Age**																		
≤53	61 (54.5)	52 (46)	0.2052	79 (71)	75 (68)	0.56	67 (56)	46 (42)		71 (45)	44 (59)	**0.039**	46 (43)	27 (59)		46 (52)	31 (52)	0.918
>53	51 (45.5)	61 (54)		32 (29)	36 (32)		53 (44)	63 (58)	**0.039**	87 (55)	30 (41)		60 (57)	19 (41)	0.08	43 (48)	28 (48)	
**Histotype**																		
IDC	91 (85)	99 (88)	**0.012**	130 (85)	60 (88)	0.20	100 (84)	95 (87)	0.55	133 (85)	65 (88)	**0.035**	87 (82)	42 (91)		75 (84)	53 (90)	0.359
ILC	4 (4)	11 (10)		9 (6)	6 (9)		8 (7)	8 (7)		15 (9)	1 (1)		7 (7)	1 (2)		6 (7)	1 (2)	
Other	12 (11)	3 (2)		14 (9)	2 (3)		11 (9)	6 (6)		9 (6)	8 (11)		12 (11)	3 (7)	0.32	8 (9)	5 (8)	
**T. size (cm)**																		
≤2.0	63 (57)	65 (59)	0.78	93 (61)	34 (51.5)	0.20	71 (61)	59 (54)	0.282	93 (60)	41 (56)	0.583	55 (53)	29 (63)		54 (61)	28 (48)	0.118
>2.0	48 (43)	46 (41)		60 (39)	32 (48.5)		45 (39)	50 (46)		62 (40)	32 (44)		49 (47)	17 (37)	0.247	34 (39)	30 (52)	
**Node**																		
Negative	70 (64)	69 (62)	0.68	96 (64)	41 (60)	0.56	81 (69)	62 (58)	0.114	95 (62)	52 (71)	0.159	66 (63)	27 (59)		58 (66)	35 (60)	0.493
Positive	39 (36)	43 (38)		53 (36)	27 (40)		37 (31)	44 (42)		59 (38)	21 (29)		38 (37)	19 (41)	0.57	30 (34)	23 (40)	
**Grade**																		
1-2	55 (49.6)	64 (57)	0.25	77 (50.3)	39 (58)	0.28	57 (48)	63 (58)	0.115	90 (57)	33 (45)	0.086	60 (58)	25 (54)		61 (74)	21 (36)	**<0.0001**
3	56 (50.4)	48 (43)		76 (49.7)	28 (42)		62 (52)	45 (42)		67 (43)	40 (55)		44 (42)	21 (46)	0.703	21 (26)	37 (64)	
**ER (%)**																		
<1	58(52)	21 (19)		66 (44)	9 (13)		59 (50)	20 (18)	**<0.0001**	48 (31)	33 (45)	**0.033**	26 (25)	17 (37)		16 (18)	27 (46)	**0.0002**
≥1	53 (48)	90 (81)	**<0.0001**	85 (56)	59 (87)	**<0.0001**	59 (50)	88 (82)		108 (69)	40 (55)		79 (75)	29 (63)	0.126	72 (92)	31 (54)	
**PgR (%)**																		
<1	62 (56)	32 (29)		72 (48)	18 (26)		65 (55)	29 (27)	**<0.0001**	61 (39)	35 (48)	0.206	37 (35)	18 (39)		24 (27)	30 (52)	**0.0027**
≥1	49 (44)	79 (71)	**<0.0001**	79 (52)	50 (74)	**0.003**	53 (45)	79 (73)		95 (61)	38 (52)		68 (65)	28 (61)	0.647	64 (73)	28 (48)	
**Ki67 (%)**																		
<14	32 (29)	42 (38)		43 (28)	32 (47)		38 (32)	40 (37)	0.445	64 (41)	16 (22)	**0.0047**	44 (42)	12 (26)		44 (50)	12 (21)	**0.0004**
≥14	79 (71)	69 (62)	0.15	108 (72)	36 (53)	**0.007**	80 (68)	68		92 (59)	57 (78)		61 (58)	34 (74)	**0.064**	44 (50)	46 (79)	
**HER2**																		
Negative	96 (86)	95 (85)	0.84	130 (86)	58 (85)	0.87	104 (88)	92 (85)	0.5	134 (86)	64 (86)	0.84	93 (86)	38 (83)	0.31	79 (90)	48 (83)	0.21
Positive	15 (14)	16 (15)		21 (14)	10 (15)		14 (12)	16 (15)		22 (14)	10 (14)		12 (14)	8 (17)		9 (10)	10 (17)	
**Protein Expression**													***Gene Alteration***					
	**NLRP3**			**PYCARD**			**Cyclin D1**			**MYC**			***CCND1***			***Myc***		
	**Negative**	**Positive**		**Negative**	**Positive**		**Negative**	**Positive**		**Negative**	**Positive**		**Negative**	**Positive**		**Negative**	**Positive**	
	**N (%)**	**N (%)**	***p***	**N (%)**	**N (%)**	***p***	**N (%)**	**N (%)**	***p***	**N (%)**	**N (%)**	***p***	**N (%)**	**N (%)**	***p***	**N (%)**	**N (%)**	***p***
**Luminal A**																		
no	81 (73)	73 (72)	0.24	113 (75)	39 (57)	**0.009**	83 (70)	72 (66)	0.55	99 (63)	59 (80)	**0.01**	63 (60)	36 (78)	**0.02**	48 (55)	47 (81)	**0.001**
yes	30 (27)	38 (28)		38 (25)	29 (43)		35 (30)	36 (34)		57 (37)	15 (20)		42 (40)	10 (22)		40 (45)	11 (19)	
**Luminal B-**																		
no	71(64)	65(59)	0.40	87(58)	45(66)	0.23	77(65)	62(57)	0.22	112 (72)	31(42)	**<0.0001**	62 (59)	25 (54)	0.59	58 (66)	32 (55)	0.19
yes	40(36)	46(41)		64(42)	23(34)		41(35)	46(43)		44(28)	43(58)		43 (41)	21 (46)		30 (34)	26 (45)	
**HER2+**																		
no	96 (86)	96 (86)	1.00	131 (87)	58 (85)	0.77	104 (88)	92 (85)	0.51	135 (86)	64 (86)	0.99	93 (88)	38 (82)	0.31	79 (90)	48 (83)	0.21
yes	15 (14)	15 (14)		20 (13)	10 (15)		14 (12)	16 (15)		21 (14)	10 (14)		12 (12)	8 (18)		9 (10)	10 (17)	
**TNBC**																		
no	85 (76)	99 (89)	**0.01**	122 (81)	62 (91)	**0.052**	90 (76)	98 (91)	**0.003**	122 (78)	68 (92)	**0.01**	97 (92)	39 (85)	0.15	79 (90)	47 (81)	0.13
yes	26 (24)	12 (11)		29 (19)	6 (9)		28 (24)	10 (9)		34 (22)	6 (8)		8 (8)	7 (15)		9 (10)	11 (19)	

*p*-value of Chi-squared test for the independence of categorical variables. Bold values indicate significance. IDC: Invasive ductal carcinoma; ILC: Invasive lobular carcinoma; ER: Estrogen receptor; PgR: Progesterone receptor; HER2: Human epidermal growth factor receptor 2; NLRP3: NOD-like receptor protein 3; PYCARD: Apoptosis-Associated Speck-Like Protein Containing a Pyrin and CARD domain; *CCND1*: CyclinD1 gene.

**Table 3:** Spearman Correlation.

	NLRP3		PYCARD		CyclinD1		MYC	
	r	*p*-Value	r	*p*-Value	r	*p*-Value	r	*p*-Value
**NLRP3**			0.322	**<0.0001**	0.366	**<0.0001**	−0.127	0.059
**PYCARD**					0.285	**<0.0001**	0.003	0.957
**Cyclin-D1**							−0.037	0.577

Bold values indicate significance. NLRP3, NOD like receptor protein 3; PYCARD, Apoptosis-Associated Speck-Like Protein Containing a Pyrin and CARD domain.

**Table 4:** Coupled biomarkers and clinicopathological characteristics.

	(A) Protein expression						(B) Gene expression					
	NLRP3/Cyclin D1			PYCARD/Cyclin D1			*CCND1*			*MYC*		
	Negative	Positive		Negative	Positive		Negative	Positive		Negative	Positive	
	N (%)	N (%)	*p*	N (%)	N (%)	*p*	N (%)	N (%)	*p*	N (%)	N (%)	*p*
**Age**												
≤53	42 (64)	29 (45)		51 (57)	20 (44)		24 (53)	13 (68)		29 (47,5)	4 (50)	
>53	24 (36)	36 (55)	**0.0289**	39 (43)	25 (56)	0.180	21 (47)	6 (32)	0.264	32 (52,5)	4(50)	0.895
**Histotype**												
IDC	55 (85)	58 (89)		76 (85)	41 (91)		35 (78)	19 (100)		49 (80)	8 (100)	
ILC	2 (3)	5 (8)		3 (4)	2 (4,5)		2 (4)	0		2 (3)	0	
Other	8 (12)	2 (3)	0.083	10 (11)	2 (4,5)	0.418	8 (18)	0	0.08	10 (17)	0	0.385
**T. size (cm)**												
≤2.0	38 (58,5)	34 (52)		56 (63)	22 (49)		20 (45)	9 (47)		34 (57)	3 (37,5)	
>2.0	27 (41,5)	31 (48)	0.48	33 (37)	23 (51)	0.119	24 (55)	10 (53)	0.888	26 (43)	5 (62,5)	0.306
**Node**												
Negative	46 (71)	39 (61)		59 (67)	25 (56)		28 (65)	8 (42)		38 (64)	2 (25)	
Positive	19 (29)	25 (39)	0.238	29 (33)	20 (44)	0.193	15 (35)	11 (58)	0.09	21 (36)	6 (75)	** 0.033 **
** Grade **												
1-2	28 (43)	35 (55)		38 (43)	23 (52)		25 (57)	9 (47)		32 (53)	2 (25)	
3	37 (57)	29 (45)	0.187	51 (57)	21 (48)	0.297	19 (43)	10 (53)	0.489	28 (47)	6 (75)	0.132
**ER (%)**												
<1	22 (34)	7 (11)		27 (31)	3 (7)		6 (14)	2 (10,5)		10 (17)	0	
≥1	43 (66)	58 (89)	**0.0016**	61 (69)	42 (93)	**0.0017**	38 (86)	17 (89,5)	0.733	50 (83)	8 (100)	0.211
**PgR (%)**												
<1	26 (40)	14 (22)		31 (35)	10 (22)		9 (20,5)	3 (16)		13 (22)	0	
≥1	39 (60)	50 (78)	**0.026**	57 (65)	35 (78)	0.124	35 (79,5)	16 (84)	0.665	47 (78)	8 (100)	0.143
**Ki67 (%)**												
<14	16 (25)	22 (34)		23 (26)	20 (44)		19 (43)	4 (21)		20 (33)	3 (37,5)	
≥14	49 (75)	42 (66)	0.224	65 (74)	25 (56)	**0.032**	25 (57)	15 (79)	0.094	40 (67)	5 (62,5)	0.815
**HER2**												
Negative	58 (89)	55 (86)		76 (86)	37 (82)		42 (95)	16 (84)		53 (88)	6 (75)	
Positive	7 (11)	9 (14)	0.570	12 (14)	8 (18)	0.527	2 (5)	3 (16)	0.129	7 (12)	2 (25)	0.295

**Table 5:** Univariate analysis of PFS (progression-free survival) and OS (overall survival).

	**PFS**					**OS**				
	**N. pts**	**Events**	**5-years DFS**	***p*-value**	**HR (95% CI)**	**N pts**	**Events**	**5-years OS**	***p*-value**	**HR (95% CI)**
Overall										
**NLRP3**				0.050					0.441	
0	107	23	85.9		1.00	107	8	93.5		1.000
1	111	12	93.7		1.997 (0.987–4.040)	111	5	98.2		1.554 (0.501–4.821)
**PYCARD**				**0.017**					0.059	
0	149	32	85.8		1.00	149	13	93.3		1.000
1	66	5	93.9		2.974 (1.158–7.637)	66	1	98.5		5.650 (0.737–43.318)
**Ciclina D1**				0.909					0.560	
0	117	21	88.9		1.00	117	7	94.9		1.000
1	106	17	87.6		1.038 (0.546–1.975)	106	9	94.3		0.739 (0.266–2.054)
**MYC**				0.779					**0.041**	
0	155	25	87.1		1.00	155	7	97.4		1.000
1	72	13	91.6		0.909 (0.465–1.776)	72	8	90.3		0.347 (0.120–1.005)
**CCND1**				0.227					**0.013**	
0	102	12	94.1		1.00	102	2	98.0		1.000
1	43	8	88.1		0.580 (0.237–1.419)	43	5	93.0		0.162 (0.031–0.836)
**Myc**				**0.017**					0.378	
0	89	7	96.6		1.00	89	3	97.8		1.000
1	55	12	85.5		0.339 (0.133–0.864)	55	4	96.4		0.513 (0.113–2.327)
**NLRP3/CCND1**				0.155					0.064	
0-0/0	43	8	90.7		1.00	43	2	95.3		1.000
1-1/1	19	4	84.2		0.642 (0.263–1.567)	19	2	100		2.227 (0.314–15.810)
2-1/0	54	3	98.1		0.482 (0.156–1.486)	54	0	100		-
3-0/1	23	4	91.3		1.168 (0.483–2.825)	23	3	87.0		2.983 (0.498–17.856)
**NLRP3/** **Ciclina D1**				0.370					0.116	
0-0/0	63	13	88.9		1.00	63	2	96.8		1.000
1-1/1	65	8	92.3		1.221 (0.367–4.062)	65	3	98.5		1.557 (0.252–9.644)
2-1/0	42	4	95.2		0.280 (0.074–1.055)	42	2	97.6		1.700 (0.235–12.269)
3-0/1	39	8	81.7		0.995 (0.299–3.304)	39	6	87.2		4.886 (0.957–24.938)
**PYCARD/CCND1**				0.242					0.093	
0-0/0	58	9	93.1		1.00	58	2	96.6		1.000
1-1/1	8	2	75.0		0.471 (0.158–1.403)	8	1	100		3.629 (0.329–40.028)
2-1/0	38	2	97.4		0.259 (0.034–1.948)	38	0	100		-
3-0/1	34	6	91.0		1.290 (0.625–2.660)	34	4	91.2		3.549 (0.650–19.377)
**PYCARD/Ciclina D1**				0.141					0.133	
0-0/0	87	17	88.5		1.00	87	6	94.3		1.000
1-1/1	44	4	93.2		1.971 (0.424–9.174)	44	1	97.7		0.355 (0.042–2.978)
2-1/0	19	1	94.7		0.324 (0.070–1.502)	19	0	100		-
3-0/1	57	13	82.1		1.206 (0.429–3.390)	57	7	89.5		1.966 (0.655–5.895

Bold values indicate significance. HR, Hazard-ratio; IDC, Invasive ductal carcinoma; ILC, Invasive lobular carcinoma; ER, Estrogen receptor; PR, Progesterone receptor; HER2, Human epidermal growth factor receptor 2; NLRP3, NOD like receptor protein 3; PYCARD, Apoptosis-Associated Speck-Like Protein Containing a Pyrin and CARD domain.; *CCND1*: CyclinD1 gene.

**Table 6:** Multivariate analysis of PFS.

					**95.0% Cl Per Exp(B)**	
	**B**	**SE**	***p*-Value**	**Exp(B)**	**Lower**	**Upper**
**PYCARD**	0.847	0.842	0.314	2.333	0.448	12.147
**NLRP3**	0.679	0.728	0.351	1.973	0.474	8.213
**CyclinD1**	−0.932	0.785	0.235	0.394	0.085	1.832
**MYC**	−0.911	0.705	0.196	0.402	0.101	1.600
***CCND1***	−1.130	0.683	0.098	0.323	0.085	1.231
***MYC* gene**	−1.677	0.800	0.036	0.187	**0.039**	0.897

Bold values indicate significance. HR, Hazard-ratio; NLRP3, NOD-like receptor protein 3; PYCARD, Apoptosis-Associated Speck-Like Protein Containing a Pyrin and CARD domain; *CCND1*: CyclinD1 gene.

## Data Availability

Data is contained within the article and supplementary material.

## Supplementary Materials

The following supporting information can be downloaded at: https://www.mdpi.com/article/10.3390/ph15060651/s1, Figure S1: Kaplan–Maier curve analysis; Figure S2: Kaplan–Maier curve analysis in luminal phenotypes. Table S1: Characteristics, dilutions and localization of used antibodies.
