# Supplementary material for: Downstream Signaling of Inflammasome Pathway Affects Patients’ Outcome in the Context of Distinct Molecular Breast Cancer Subtypes
Source: Pharmaceuticals (Basel). 2022 May 24;15(6):651. doi: 10.3390/ph15060651 (PMC9229152; doi:10.3390/ph15060651)
Supplement: Supplementary file 1 [file pharmaceuticals-15-00651-s001.zip › pharmaceuticals-1686801-supplementary.pdf]

Table S1. Characteristics, diluitions and localization of used antibodies

Antibody target	Antibody type	Antigen retrieval buffer	Dilution	Supplier	Code	Localization
NLRP3	Rabbit polyclonal	EDTA	1:50	abcam	Ab214185	cytoplasm
Pycard	Mouse monoclonal	Citrate	1:600	ThermoFisher	MA5-26363	cytoplasm
Cyclin D1						
MYC						

Citrate buffer at pH 6; ethylenediaminetetraacetic acid (EDTA) at pH 7.8. NLRP3: NOD-like receptor protein 3; PYCARD: Apoptosis-Associated Speck-Like Protein Containing a Pyrin and CARD domain;

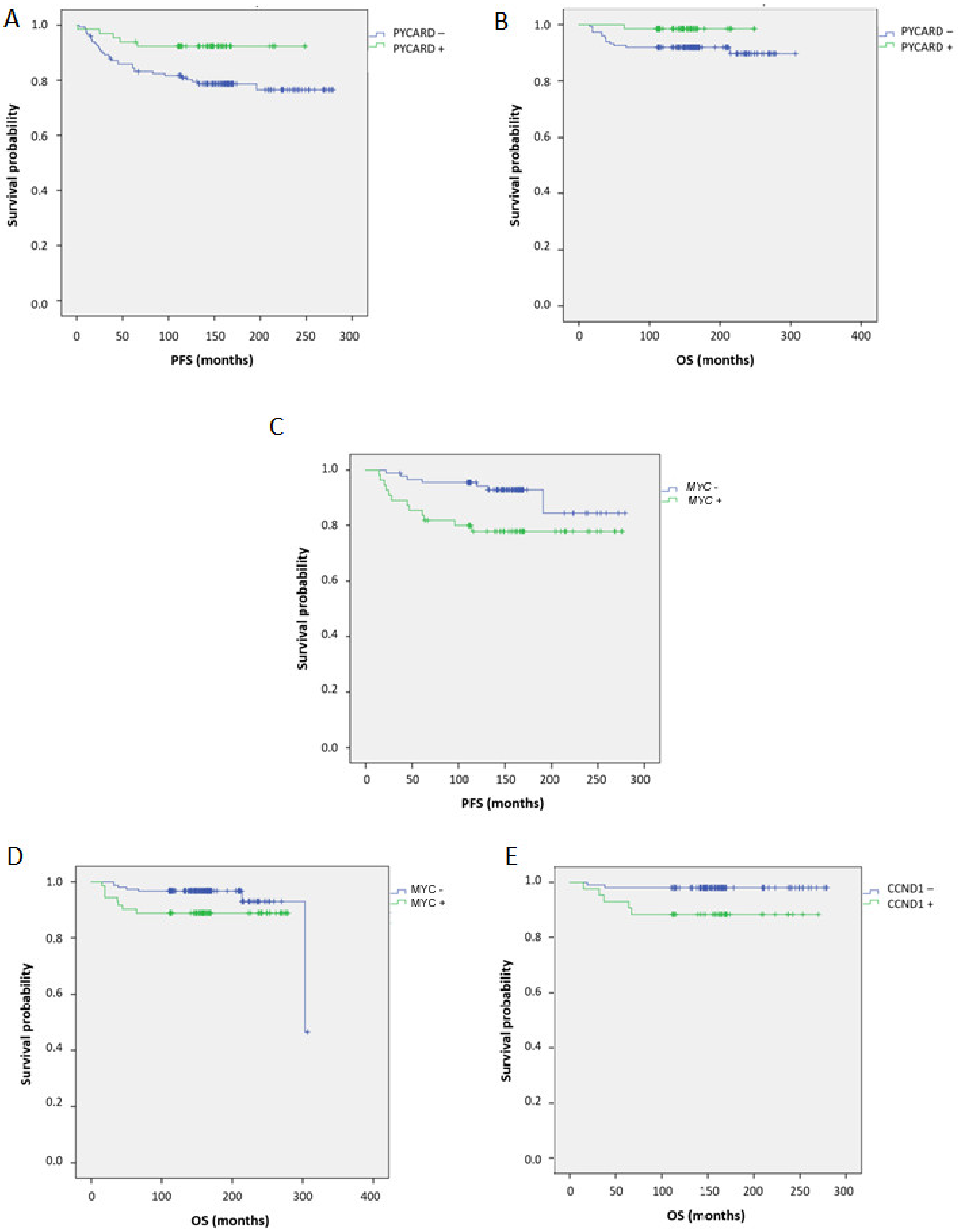

Figure S1. Kaplan–Maier curve analysis. (A) Kaplan–Maier curve for PFS according to PYCARD positive versus negative patients(p=0.017). (B) Kaplan–Maier curve for OS according to PYCARD positive versus negative patients (p=0.059). (C) Kaplan–Maier curve for PFS according to *MYC* gene positive versus negative patients (p=0.017). (D) Kaplan–Maier curve for OS according to MYC positive versus negative patients(p=0041) (E) Kaplan–Maier curve for OS according to *CCND1* positive versus negative patients(p=0.013).

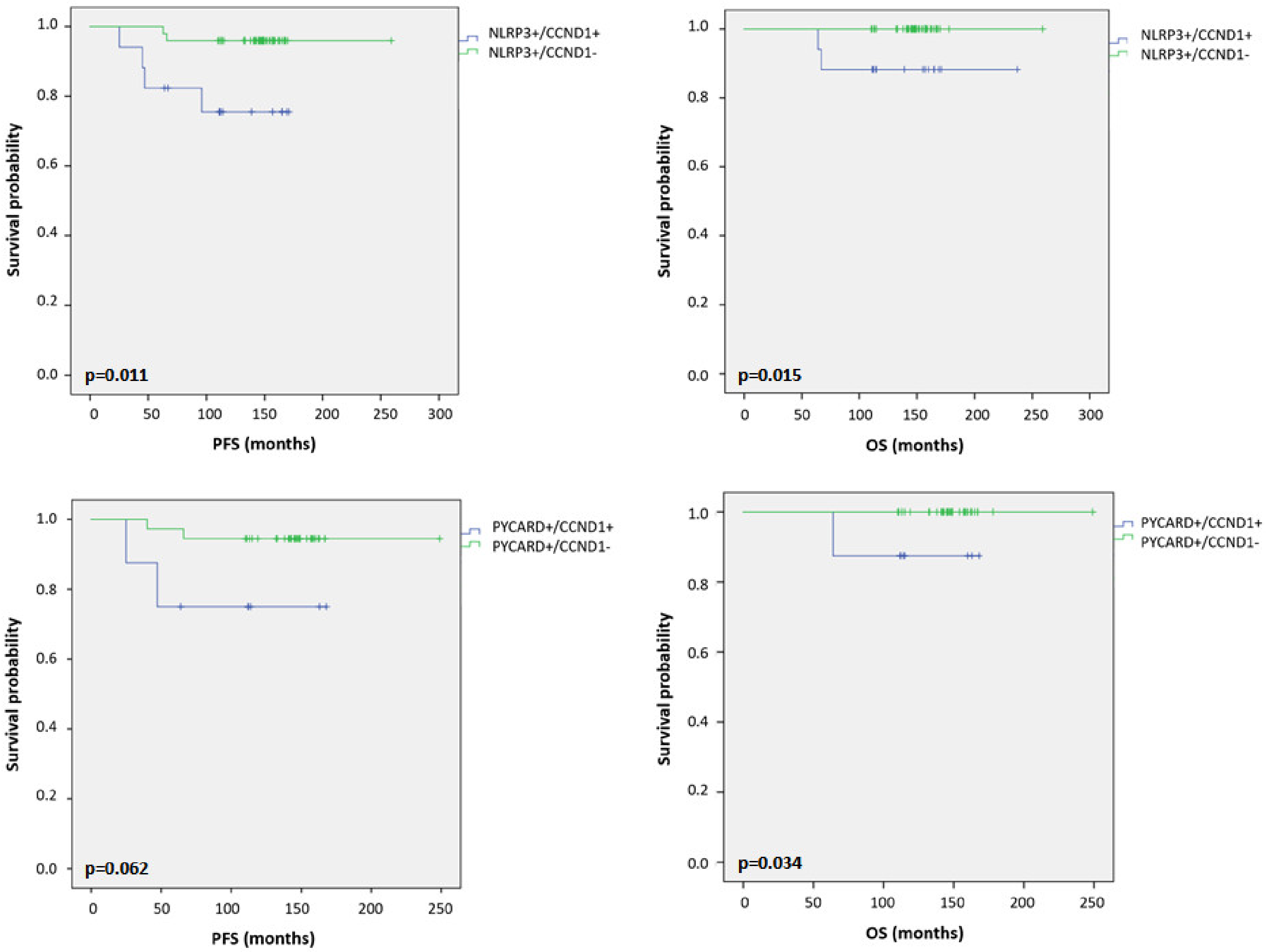

Figure S2. Kaplan–Maier curve analysis in Luminal phenotypes. (A) Kaplan–Maier curve for progression-free survival (PFS) according to NLRP3+/*CCND1*+ versus NLRP3+/*CCND1*- patients (p=0.011). (B) Kaplan–Maier curve for overall survival (OS) according to NLRP3+/*CCND1*+ versus NLRP3+/*CCND1*- patients (p=0.015). (C) Kaplan–Maier curve for PFS according to PYCARD+/*CCND1*+ versus PYCARD+/*CCND1*- patients (p=0.062). (D) Kaplan–Maier curve for OS according to PYCARD+/*CCND1*+ versus PYCARD+/*CCND1*- patients (p=0.034).
